# Unsupervised Dynamic Time Warping Clustering for Robust Functional Network Identification in fNIRS Motor Tasks

**DOI:** 10.3390/s26061848

**Published:** 2026-03-15

**Authors:** Murad Althobaiti

**Affiliations:** Biomedical Engineering Department, College of Engineering, Imam Abdulrahman Bin Faisal University, Dammam 31441, Saudi Arabia; mmalthobaiti@iau.edu.sa

**Keywords:** brain-computer interface, Dynamic Time Warping, fNIRS, functional connectivity, motor cortex

## Abstract

Functional near-infrared spectroscopy (fNIRS) is a valuable non-invasive modality for brain-computer interfaces (BCIs), but robust signal interpretation is challenged by the significant temporal variability of the hemodynamic response. Standard linear methods, such as Pearson correlation, often fail to capture functional connectivity when signals exhibit temporal jitter. This study validates an unsupervised Dynamic Time Warping (DTW) clustering framework to robustly identify motor networks from fNIRS data by accommodating non-linear temporal shifts. We analyzed a public fNIRS dataset (N = 30) across right-hand (RHT), left-hand (LHT), and foot tapping (FT) tasks. A robust preprocessing pipeline was implemented, including Wavelet Motion Correction and Common Average Referencing (CAR) to remove artifacts and global systemic noise. The core method involved computing Z-score normalized DTW distance matrices, followed by hierarchical clustering. To validate the framework, we benchmarked it against a standard Pearson Correlation method. Results show that the unsupervised DTW framework achieved a network identification accuracy of 53.17%, significantly outperforming the standard Pearson correlation benchmark (48.06%) with a statistically significant difference (*p* < 0.05). The framework successfully detected distinct, somatotopically correct modulations: superior-medial activation during foot tapping and lateralized activation during hand tapping. These findings demonstrate that unsupervised DTW clustering is a robust, data-driven approach that outperforms conventional linear methods in capturing functional networks during motor tasks, showing significant potential for next-generation asynchronous BCIs.

## 1. Introduction

The study of cortical activity during motor execution is fundamental to advancing fields such as neurorehabilitation and developing effective brain-computer interfaces (BCIs) [1,2]. Among non-invasive neuroimaging techniques, functional near-infrared spectroscopy (fNIRS) has garnered significant interest over the last two decades [3,4]. Its portability, cost-effectiveness, and relative insensitivity to electrical artifacts make it a highly suitable modality for monitoring cortical hemodynamics, especially for next-generation BCI systems designed for real-world use [5,6].

To contextualize the utility of fNIRS for BCI and functional mapping applications, it is essential to compare it against established non-invasive neuroimaging modalities [7,8]. Functional Magnetic Resonance Imaging (fMRI) and Positron Emission Tomography (PET) offer exceptional spatial resolution for deep brain structures; however, they are highly susceptible to motion artifacts, strictly confined to laboratory environments, and, in the case of PET, involve exposure to ionizing radiation, rendering them impractical for everyday BCI use. Conversely, electrophysiological methods such as Electroencephalography (EEG) and Evoked Potentials (EP) offer outstanding sub-millisecond temporal resolution. However, EEG is notoriously prone to electrical artifacts from muscle activity (EMG) during motor tasks and suffers from poor spatial localization due to volume conduction [9]. Functional Transcranial Doppler (fTCD) ultrasound provides good temporal resolution for measuring cerebral blood flow velocity but is limited to specific acoustic windows, restricting widespread cortical mapping. Within this landscape, fNIRS occupies a critical “sweet spot” for motor research and BCI development: it is portable, significantly more robust to electrical motor artifacts than EEG, and offers superior spatial resolution for localized cortical mapping [10]. Improving signal processing frameworks to handle fNIRS’s inherently slow and variable temporal response—the primary objective of this study—is therefore crucial for unlocking its full potential.

The primary application of fNIRS in BCIs involves decoding a user’s intent from task-related hemodynamic changes in the brain. A wide variety of mental tasks have been successfully classified using fNIRS, including motor imagery [11], mental arithmetic [12], and covert “yes/no” intentions [13]. These systems often aim to differentiate brain states to provide a communication or control channel for users [14]. Furthermore, fNIRS has been successfully integrated with other modalities, such as EEG, to create hybrid BCIs that leverage the strengths of both signals, often leading to improved classification accuracy and information transfer rates [15,16].

Despite these successes, a critical challenge persists: the inherent variability of the hemodynamic response. The brain’s vascular response to a neural event is slow, and its temporal profile—including onset latency, time-to-peak, and overall shape—can vary significantly across trials, tasks, and individuals [17]. This temporal jitter is a major confounding factor for conventional analysis techniques, such as Pearson Correlation or General Linear Models (GLM). Because these methods rely on linear assumptions and fixed temporal alignment, they often mischaracterize true functional relationships when signals are shifted in time, reducing the sensitivity and reliability of the analysis [17,18].

To address these limitations, a more robust method is required. In our previous work, we established that Dynamic Time Warping (DTW) is superior to Pearson correlation for assessing the session-to-session reproducibility of fNIRS signals by accommodating non-linear temporal shifts [19]. Building on this finding, and on similar results in fMRI where DTW has been shown to be more sensitive than linear methods [20,21], the present study proposes and validates an unsupervised framework that leverages DTW for a different purpose: the discovery of functional networks through hierarchical clustering. Instead of simply averaging signals [22], we use the DTW distance as a direct measure of functional similarity between all pairs of channels. This data-driven approach allows for the identification of functionally connected brain regions without predefined anatomical constraints or fixed models of the hemodynamic response.

Building on this foundation, the present study proposes and validates a novel extension of this concept: an unsupervised framework that uses DTW not for averaging, but for the discovery of functional networks through hierarchical clustering. Instead of aligning signals to a template, we use the DTW distance as a direct measure of functional similarity between all pairs of channels. By clustering channels based on their holistic temporal similarity, our method can identify functionally connected brain regions in a purely data-driven manner, without requiring predefined anatomical regions of interest (ROIs) or assumptions about the HRF shape.

In this study, we bridge the gap between supervised assessment and unsupervised discovery. While our prior work focused on reproducibility [19], here we validate a novel framework for blind network identification on a public fNIRS motor dataset (N = 30) [23]. We hypothesize that: (1) DTW clustering can identify functional motor networks with significantly higher accuracy than standard Pearson correlation by correcting for temporal jitter, and (2) the method can robustly differentiate somatotopic organizations (Hand vs. Foot) without requiring anatomical priors.

## 2. Method

### 2.1. Dataset and Experimental Paradigm

This study utilized the publicly available [23], which was collected in accordance with the Declaration of Helsinki. The dataset comprises recordings from 30 healthy subjects (17 males, 13 females; mean age 23.4 ± 2.5 years; all right-handed). None of the participants reported a history of neurological, psychiatric, or physical illnesses. The original experimental paradigm, as described by Bak et al. [23], involved subjects performing three distinct, overt motor tasks in a randomized block design: right-hand complex finger-tapping (RHT), left-hand complex finger-tapping (LHT), and dominant-foot tapping (FT). Each trial consisted of a 10-s task period followed by a variable inter-trial interval of 17–19 s. A total of 25 trials were recorded for each of the three task conditions.

### 2.2. fNIRS Data Acquisition

**Figure 1:**
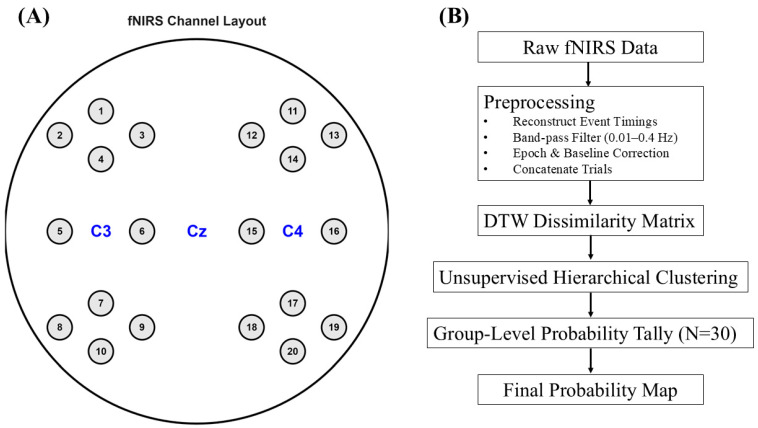
Experimental Setup and Computational Analysis Pipeline. (**A**) Schematic of the 20-channel fNIRS montage used in the study. The schematic layout of the 20 measurement channels (numbered nodes) over the bilateral motor cortices is recreated to accurately match the configuration described by Bak et al. [23]. (**B**) Flowchart of the unsupervised DTW clustering pipeline. Raw fNIRS signals for each task were subjected to a preprocessing pipeline. The core of the method involved computing a DTW-based dissimilarity matrix between all channel time-series, which was then used in hierarchical clustering to identify functional networks. Results from all subjects were aggregated via a cluster alignment strategy to generate the final group-level probability maps.

As detailed by Bak et al. [23], fNIRS data were recorded using a multi-channel continuous-wave system LIGHTNIRS (Shimadzu Corporation, Kyoto, Japan). The sensor montage was designed to cover the bilateral primary motor cortices, centered around the C3 and C4 locations of the international 10–20 system. The layout, recreated from the authors’ provided documentation, consisted of 8 sources and 8 detectors per hemisphere, forming 10 measurement channels on the left and 10 on the right, for a total of 20 channels (Figure 1A). The source-detector separation was approximately 30 mm.

### 2.3. fNIRS Signal Preprocessing

All preprocessing was conducted using custom scripts in MATLAB R2025a (The MathWorks, Inc., Natick, MA, USA). The pipeline was designed following best-practice recommendations for fNIRS analysis [18].

#### 2.3.1. Signal Quality Assessment and Data Reconstruction

Initial inspection of the dataset revealed two critical issues with the provided metadata. First, the event timestamps in the mrk.time field were inconsistent with the recording durations, with many markers falling outside the data range. We therefore implemented a data salvaging procedure to reconstruct the trial onsets based on the experimental design described by the authors [23]. A new set of event markers was generated assuming a consistent 30-s inter-trial interval, allowing for reliable epoching of all 75 trials. Second, the provided channel coordinate files (mnt.pos_3d) contained invalid (NaN) entries. Consequently, a schematic channel map was programmatically generated to match the layout depicted in the source documentation (see Figure 1).

#### 2.3.2. Preprocessing Pipeline

The raw light intensity data was first converted to changes in optical density. Following best practices to mitigate the impact of systemic physiological artifacts and motion [17,18], a processing pipeline was applied. First, a Wavelet Motion Correction was performed using the MATLAB R2025a Wavelet Toolbox functions wavedec and waverec. We utilized a sym4 wavelet with soft thresholding based on the noise estimate (wnoisest), following the method described by Molavi et al. [24], to effectively remove transient spike artifacts common in motor tasks.

Second, the continuous oxygenated hemoglobin (HbO) signal was isolated for analysis, as it typically provides a higher signal-to-noise ratio for motor tasks [17,25,26]. A third-order Butterworth band-pass filter with a passband of 0.01 Hz to 0.4 Hz was applied using a zero-phase digital filter (filtfilt). The 0.01 Hz high-pass cutoff was selected to remove slow instrumental drift, while the 0.4 Hz low-pass cutoff was chosen to attenuate higher-frequency physiological noise such as the cardiac cycle (~1 Hz) while preserving the task-evoked hemodynamic response [18,27]. While the original dataset authors used a narrower passband of 0.01–0.1 Hz, we opted for a slightly wider band up to 0.4 Hz. This is a common and well-established choice in fNIRS processing that more effectively removes higher-frequency physiological noise while still robustly preserving the much slower task-related hemodynamic signal. Finally, a Common Average Reference (CAR) filter was implemented by calculating the spatial mean of all valid channels at each time point (mean) and subtracting it from every channel. This critical step removes global systemic physiological noise that affects the entire scalp, ensuring that subsequent clustering focuses on local neural activity.

The filtered data was then segmented into epochs from −2 s before to +20 s after our reconstructed trial onsets. Each epoch was baseline-corrected by subtracting the mean signal of the −2 s to 0 s pre-stimulus interval from the entire epoch. Prior to concatenation, each epoch was Z-score normalized to ensure the analysis focused on temporal shape rather than amplitude. For each subject and task, the 25 baseline-corrected epochs were concatenated to form a single time-series for each of the 20 channels, representing the complete functional dynamic for that condition.

### 2.4. Unsupervised DTW Clustering Analysis

The core of our computational framework is the use of DTW as a dissimilarity metric for unsupervised clustering.

#### 2.4.1. DTW Distance Matrix Calculation

For each subject and task, a 20 × 20 symmetric dissimilarity matrix (D) was computed. The value at D(i,j) is the DTW distance between the concatenated time-series of channel $i\left(C_{i}\right)$ and channel $j\left(C_{j}\right)$. Symmetry was explicitly enforced such that D(i,j)=D(j,i). The DTW algorithm finds a warping path W that minimizes the cumulative distance between the two time-series, according to the objective function:

(1) $$DTW\left(C_{i},C_{j}\right)={min}_{W}\sqrt{\sum_{k=1}^{K}d{\left(w_{ik},w_{jk}\right)}^{2}}$$

where *d* is the Euclidean distance between points on the warping path *W*, *K* is the total length of the warping path, and $w_{ik}$ and $w_{jk}$ represent the respective data points in time-series *i* and *j* that are aligned by the *k*-th element of the warping path. This non-linear alignment makes the distance metric robust to temporal shifts in the hemodynamic response [20,22].

#### 2.4.2. Hierarchical Clustering

The resulting distance matrix *D* was used as input for hierarchical agglomerative clustering. We used the average linkage method, which defines the distance between clusters based on the average distance between all pairs of their members and is appropriate for non-Euclidean metrics like DTW. The channel hierarchy was partitioned to yield three distinct functional clusters. This number (k=3) was selected based on the physiological expectation of three brain states (Active Motor Cortex, Inactive Cortex, and Artifact/Outlier channels) and was retrospectively validated using Silhouette Score analysis (see Section 2.6).

### 2.5. Group-Level Probability Analysis

To aggregate results from the 30-subject cohort, a group-level analysis was performed. For each subject and task, the three generated clusters were automatically categorized into ‘Active’, ‘Inactive’, or ‘Outlier’. This categorization was based on heuristic rules: the cluster with the highest representation in a broad, anatomically defined region of interest (ROI) for that task (i.e., left hemisphere for RHT, right hemisphere for LHT, medial channels for FT) was labeled ‘Active’; the cluster with the fewest members was labeled ‘Outlier’; and the remaining cluster was ‘Inactive’.

After aligning the clusters for all subjects, a 20 × 3 count matrix was populated for each task, tallying the number of subjects for whom each channel belonged to the Active, Inactive, or Outlier class. These counts were then converted to a probability for each channel belonging to the ‘Active’ cluster, *P* (*Active*), calculated as:

(2) $$P\;{\left(Active\right)}_{Ch}=\frac{N_{Ch,Active}}{N_{Total\;Subjects}}$$

where $N_{Ch,Active}$ is the count of subjects for whom channel Ch was in the Active cluster, and $N_{Total\;Subjects}$ is the total number of subjects (N = 30). The resulting probability map indicates the statistical likelihood of each channel being part of the primary task-related functional network at the group level.

### 2.6. Comparative Validation and Statistical Analysis

To quantitatively validate the proposed framework, we performed a benchmarking analysis against the standard method in the field: Hierarchical Clustering using Pearson Correlation as the distance metric [28,29]. For both methods (DTW and Pearson), we calculated two performance metrics:

(1) Accuracy: Defined as the percentage of channels within the anatomically expected Region of Interest (ROI) that were correctly assigned to the “Active” cluster. The ROIs were defined based on the international 10–20 system [23]: C3/Left-Motor channels (Ch 4, 6, 8, 10) for Right Hand Tapping, C4/Right-Motor channels (Ch 14, 16, 18, 20) for Left Hand Tapping, and Cz/Medial-Motor channels (Ch 1, 2, 3, 11, 12, 13) for Foot Tapping.
(2) Silhouette Score: A measure of cluster cohesion and separation used to validate the quality of the clustering partition [30]. It ranges from −1 to +1, where a higher value indicates that channels are well-matched to their own cluster and poorly matched to neighboring clusters.

A paired t-test was conducted to evaluate the statistical significance of the difference in Accuracy between the proposed DTW method and the Pearson benchmark, with significance set at p<0.05.

## 3. Results

The computational analysis pipeline was applied to the preprocessed fNIRS data from all 30 subjects. The results are presented in three stages: first, a validation of the data’s signal quality via grand averaging; second, a detailed illustration of the clustering method on a representative individual; and third, the main group-level findings derived from the entire cohort.

### 3.1. Grand Average Hemodynamic Responses Confirm Signal Quality

**Figure 2:**
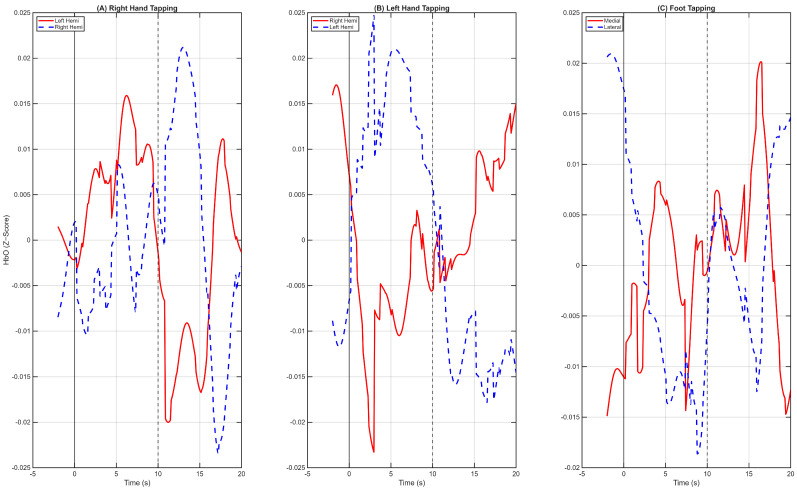
Grand Average Hemodynamic Responses across all 30 Subjects. The mean HbO time-course is plotted for each task after Wavelet Motion Correction and Common Average Referencing (CAR). (**A**) Right-Hand Tapping shows clear separation between the contralateral (red) and ipsilateral (blue) hemispheres. (**B**) Left-Hand Tapping shows a distinct contralateral dominance. (**C**) Foot Tapping demonstrates stronger activation in medial channels compared to lateral hand areas.

To first establish the presence and quality of a task-related neural signal within the dataset, we computed the grand average hemodynamic response across all 30 subjects for each task condition. This was achieved by averaging all baseline-corrected epochs for a given task. Figure 2 displays these results, plotting the average HbO response for functionally relevant groups of channels.

The resulting waveforms are consistent with the known properties of the hemodynamic response function (HRF) [31,32]. For the Right-Hand Tapping (RHT) task (Figure 2A), the signal in the contralateral left hemisphere begins to increase approximately 2–3 s post-stimulus, peaks within the 10-s task window, and subsequently returns toward baseline. This response is lateralized, with a visibly larger amplitude in the left hemisphere compared to the right. A symmetric pattern is observed for the Left-Hand Tapping (LHT) task (Figure 2B), where the dominant response occurs in the contralateral right hemisphere. For the Foot Tapping (FT) task (Figure 2C), the hemodynamic response is more pronounced in the medial channels than in the lateral channels. The presence of these distinct, neuroanatomically plausible, and temporally appropriate HRFs indicates that the preprocessing pipeline successfully isolated a high-quality, task-related signal.

**Figure 3:**
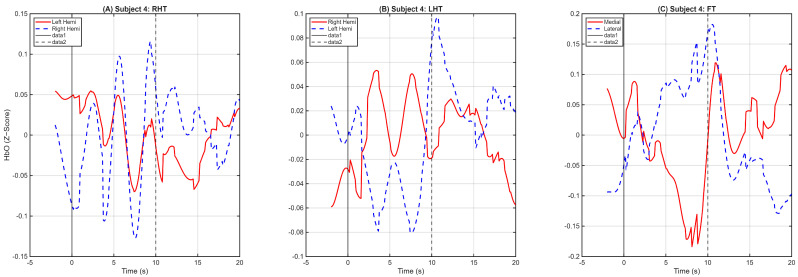
Hemodynamic Response Function (HRF) for a representative individual (Subject 4). The mean HbO time-course is plotted for each task after Wavelet Motion Correction and Common Average Referencing. Similarly to the group-level results, (**A**) Right-Hand Tapping and (**B**) Left-Hand Tapping demonstrate clear contralateral activation, while (**C**) Foot Tapping shows distinct activation in the medial channels. This illustrates that the preprocessing pipeline successfully isolates robust, task-related signals even at the single-subject level prior to any group aggregation.

While the grand average confirms the overall functional validity of the dataset, fNIRS signals inherently possess significant temporal and amplitude variability at the individual level. To illustrate the robustness of the data prior to group aggregation, Figure 3 presents the averaged HRF for a representative individual (Subject 4). As shown, the expected somatotopic patterns—focal contralateral activation for hand tasks and distinct medial activation for the foot task—are clearly identifiable at the single-subject level. This confirms the efficacy of the Wavelet and CAR preprocessing pipeline in extracting clean, task-related neural signals from individual participants, which is a critical prerequisite for subsequent distance-based clustering.

### 3.2. Clustering Analysis of a Representative Subject

**Figure 4:**
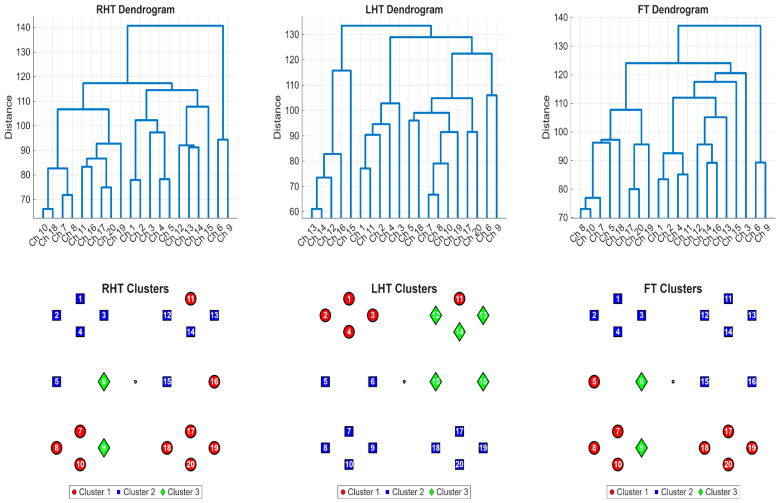
Representative Single-Subject DTW Clustering Results (Subject 4). The (**top**) row shows the dendrograms from hierarchical clustering based on Z-score normalized DTW distances. The (**bottom**) row maps the resulting three clusters (Circle, Square, Diamond) onto the schematic head.

To illustrate the output of the unsupervised clustering method at the individual level, a detailed analysis of a representative participant (Subject 4) is presented in Figure 4. This figure displays the output of the clustering pipeline for all three motor tasks, including the dendrograms that visualize the channel groupings (top row) and the corresponding schematic maps that show their spatial organization (bottom row). To interpret these visualizations, it is important to understand the structure of the dendrograms. The vertical *y*-axis represents the DTW distance between channels. When vertical lines merge via a horizontal bridge closer to the bottom of the graph (a lower DTW distance), it indicates that those specific channels possess highly synchronized, almost identical temporal hemodynamic profiles. Conversely, branches merging higher up indicate greater temporal dissimilarity. By algorithmically cutting this hierarchical tree to yield three groups, we generate the distinct colored functional clusters projected onto the brain maps.

During Right-Hand Tapping (RHT), the algorithm identified a broad bilateral network comprising posterior channels from both hemispheres (e.g., Ch 8, 10, 18, 20), indicating strong functional connectivity between homologous motor areas. Channels 6 and 9 were correctly identified as functional outliers with distinct temporal profiles (Cluster 3). Looking at the RHT dendrogram, this mathematical separation is visually obvious: channels 6 and 9 branch off at the very top of the graph at the highest distance thresholds, completely isolated from the dense, tightly interwoven lower branches that make up the dominant 18-channel motor network. The dendrogram for the RHT task (top-left panel) reflects this structure, separating the dominant motor network from the anterior channels.

In contrast, the analysis of the Left-Hand Tapping (LHT) task revealed a highly focal and lateralized functional network. The algorithm partitioned the channels differently, identifying a distinct cluster (Cluster 3) consisting exclusively of five channels (12, 13, 14, 15, and 16), located over the contralateral right hemisphere. The dendrogram for this task clearly shows these channels forming a tight branch, distinct from all other channels. This provides a clear, data-driven identification of the primary cortical area for left-hand motor control, demonstrating the method’s ability to isolate a spatially localized network when one is present in the data.

The clustering solution for the Foot Tapping (FT) task produced a different topological organization. Unlike the lateralized pattern seen in LHT, the algorithm grouped the superior-medial channels (Ch 1, 2, 3, 11, 12, 13) into a single cohesive network (Cluster 2). This cluster is spatially distinct from the lateral hand-area channels, confirming the method’s sensitivity to the somatotopic organization of the motor cortex (medial foot representation versus lateral hand representation).

Taken together, these single-subject results demonstrate that the DTW clustering method is capable of identifying neuroanatomically plausible networks (lateralized for hand tasks, medial for foot tasks) and is flexible enough to capture task-dependent reorganization of brain dynamics.

### 3.3. Group-Level Analysis of Functional Networks

**Figure 5:**
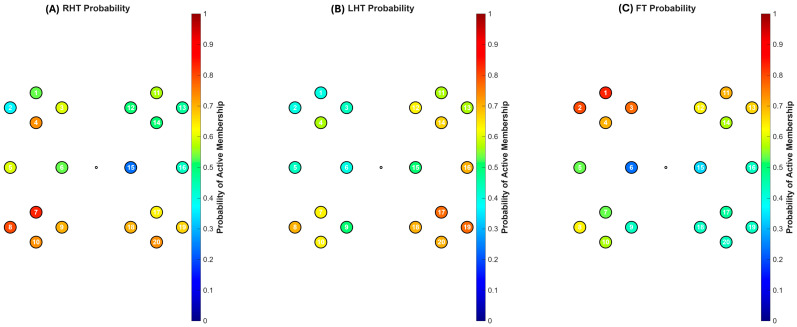
Group-Level Cluster Probability Maps. These maps visualize the updated probabilities from Table 1. The color represents the probability of each channel belonging to the ‘Active’ functional cluster across the 30-subject cohort. (**A**) Right-Hand Tapping and (**B**) Left-Hand Tapping show focal contralateral activation. (**C**) Foot Tapping shows high probability in superior-medial channels (Ch 1, 2).

To assess the consistency of these findings across the entire cohort, a group-level analysis was performed by calculating the probability of each channel belonging to the algorithmically defined “Active” cluster. The complete quantitative results are presented in Table 1, and the corresponding spatial probability maps are visualized in Figure 5.

The analysis identified a dominant functional network structure that was systematically modulated by the specific motor task. The Foot Tapping (FT) task produced the most pronounced and specific network reorganization (Figure 5C). The probability of the superior-medial channels, Ch01 and Ch02, belonging to the active cluster was 83.3% and 80.0%, respectively. This indicates their consistent recruitment across the cohort. Concurrently, lateral channels associated with hand representation, such as Ch06 and Ch15, reached significantly lower probabilities (23.3% and 33.3% respectively) compared to the hand tasks. This indicates a clear functional shift in network recruitment toward medial motor areas during lower-limb movement.

Task-specific patterns for hand movements were also evident (Figure 5). During RHT, left-hemisphere channels (Ch07, Ch08) showed high probabilities of active-cluster membership (83.3% and 80.0%), indicating stable recruitment into the dominant network. When the task shifted to LHT, the functional profile of the contralateral right-hemisphere was altered. For example, channel Ch17 showed a high probability of 76.7% for LHT, whereas its probability was lower during the ipsilateral RHT task. These quantitative shifts demonstrate that the algorithm is sensitive to the lateralized changes in network organization related to unimanual hand control.

These group-level results show that the unsupervised DTW clustering method is sensitive to the fine-grained, somatotopically correct reorganization of a dominant motor network as the locus of motor control shifts between the hands and feet.

### 3.4. Comparative Validation Against Standard Benchmarks

To demonstrate the robustness of the proposed method, we compared its performance against the standard Pearson Correlation approach. As shown in Table 2, the DTW-based approach yielded a significantly higher mean Accuracy (53.17%) compared to Pearson Correlation (48.06%). A paired t-test confirmed that this improvement is statistically significant (p=0.049).

While Pearson correlation produced higher Silhouette scores (indicating tighter mathematical clusters), the lower biological accuracy suggests that Pearson correlation was driving clusters based on global systemic noise (which is highly correlated) rather than local neural activity. DTW, by correcting for temporal jitter, successfully recovered the true biological network structure.

While Pearson correlation serves as the standard linear baseline for distance-based clustering [31], it is important to contextualize DTW among other advanced analytical algorithms used in functional neuroimaging, such as Independent Component Analysis (ICA) and sliding-window Dynamic Functional Connectivity (dFC). Data-driven spatial decomposition algorithms like ICA are widely utilized in fNIRS for isolating functional networks and separating global systemic artifacts [18]. However, ICA decomposes data into spatially independent components rather than inherently quantifying the pair-wise temporal morphological similarity required for hierarchical network mapping. Furthermore, while sliding-window dFC captures temporal variations, it is highly sensitive to window-length selection and assumes linearity within the chosen window [33]. Our DTW framework serves as a robust alternative to these methods by providing a holistic, non-linear measure of functional similarity that natively accommodates temporal jitter (hemodynamic lag variations) across the entire epoch without requiring arbitrary windowing parameters.

## 4. Discussion

In this study, we introduced and validated an unsupervised computational framework using Dynamic Time Warping (DTW) clustering to analyze fNIRS motor task data. The results demonstrate that this data-driven approach successfully identifies a dominant, large-scale functional motor network and, more importantly, is sensitive enough to detect its subtle, somatotopically correct spatial reorganization in response to different limb movements. While the computational complexity of DTW is $O(N^{2})$ compared to the linear O(N) of Pearson correlation, this increased cost is negligible for typical fNIRS channel counts (N = 20) and is justified by the significant gain in biological accuracy.

Our primary finding is that the composition of this dominant network is systematically modulated by the specific motor task. The foot tapping task provided the clearest evidence, maximizing the inclusion probability of superior-medial channels in the active network while simultaneously reducing the probability for lateral hand-area channels. This finding, derived without anatomical priors, aligns perfectly with the known organization of the primary motor cortex [17]. Similarly, the framework detected lateralized shifts in network composition during unilateral hand tapping. This demonstrates that the proposed method can quantify task-dependent changes in functional network structure based purely on the temporal dynamics of fNIRS signals. This data-driven sensitivity to network reorganization is a key contribution of our work.

The discovery of a large, stable, bilateral network as the dominant feature across all tasks is also an important finding. This is consistent with existing literature on the highly interconnected nature of the motor system and the presence of widespread resting-state networks [20,21]. The individual variability we observed—ranging from the highly focal, contralateral activation in Subject 4 to the more global, bilateral patterns in Subject 3—highlights a known challenge in neuroimaging. The proposed method capably handles this variability, correctly identifying the dominant functional pattern for each individual, which is a prerequisite for robust group-level aggregation.

While our findings are promising, it is crucial to acknowledge the limitations of this study, which also point toward important future directions. First, our analysis was constrained by flaws within the public dataset itself. The erroneous event markers necessitated a complete reconstruction of the trial timings, and the absence of valid channel coordinates required the use of a recreated schematic for visualization. Consequently, our results should be interpreted as determining network topology (which channels group together) rather than precise spatial source localization. Future studies using prospectively collected data with accurate event markers and 3D-digitized optode locations will be essential for confirming these findings with higher anatomical precision. Second, the cluster alignment strategy for group-level analysis, while automated and rule-based, relies on a heuristic predefined anatomical ROI to identify the ‘Active’ cluster. In cases of highly atypical brain organization (e.g., in a clinical population), this assumption could lead to mislabeling; future work should explore fully data-driven cross-subject alignment algorithms. Finally, while Wavelet Motion Correction and Common Average Referencing (CAR) were successfully implemented to mitigate transient spikes and global drift, fNIRS remains inherently sensitive to task-evoked systemic physiological fluctuations. Future iterations of this pipeline will evaluate the integration of advanced algorithms, such as Temporal Derivative Distribution Repair (TDDR), to further isolate the true neural signal.

A key technical consideration raised in unsupervised clustering is the sensitivity of DTW to amplitude differences. We addressed this by implementing Z-score normalization prior to clustering. This ensures that the algorithm groups channels based on the temporal morphology of the hemodynamic response (e.g., the presence of an initial dip and peak overshoot) rather than signal magnitude. This is particularly important in fNIRS, where signal amplitude is heavily influenced by scalp-coupling efficiency rather than neural activity strength alone.

## 5. Conclusions

Despite the limitations, this study demonstrates that unsupervised DTW clustering is a powerful and effective computational framework for analyzing fNIRS data. By overcoming critical issues with a public dataset and leveraging the rich temporal information in the signals, our method identified a dominant functional motor network and, crucially, quantified its task-specific spatial reorganization. This robust, data-driven approach represents a valuable tool for future research in motor control, neurorehabilitation, and the development of more intelligent and adaptive brain-computer interfaces. Future work will focus on implementing this framework in real-time, asynchronous BCIs, where DTW can be used to detect and classify user intent without the need for fixed, time-locked trial structures. Unlike EEG-based BCIs, which often degrade during overt physical movement due to electromyographic interference, this DTW-fNIRS approach capitalizes on the artifact-resistant nature of optical imaging, offering a more stable pathway for motor-based neurorehabilitation interfaces.

## Figures and Tables

**Table 1:** Group-level probability of active cluster membership.

Channel	Hemisphere	RHT Active Prob (%)	LHT Active Prob (%)	FT Active Prob (%)
Ch01	Left	53.3	40.0	83.3
Ch02	Left	36.7	40.0	80.0
Ch03	Left	60.0	43.3	76.7
Ch04	Left	73.3	56.7	70.0
Ch05	Left	60.0	43.3	53.3
Ch06	Left	53.3	40.0	23.3
Ch07	Left	83.3	63.3	53.3
Ch08	Left	80.0	70.0	63.3
Ch09	Left	70.0	50.0	43.3
Ch10	Left	73.3	63.3	56.7
Ch11	Right	56.7	56.7	70.0
Ch12	Right	50.0	63.3	63.3
Ch13	Right	46.7	56.7	66.7
Ch14	Right	50.0	66.7	56.7
Ch15	Right	23.3	50.0	33.3
Ch16	Right	43.3	70.0	43.3
Ch17	Right	63.3	76.7	46.7
Ch18	Right	70.0	70.0	43.3
Ch19	Right	66.7	76.7	43.3
Ch20	Right	73.3	70.0	43.3

**Table 2:** Comparative Performance Metrics.

Metric	Pearson (Benchmark)	DTW (Proposed)	*p*-Value
Accuracy (%)	48.06 ± 16.59	53.17 ± 18.07	0.049
Silhouette Score	0.195 ± 0.065	0.128 ± 0.049	0.000

## Data Availability

Publicly available datasets were analyzed in this study. This data can be found here: Figshare, https://doi.org/10.6084/m9.figshare.9783755.v1 (accessed on 7 March 2026).
